# Impact of Predator Exclusion and Habitat on Seroprevalence of New World *Orthohantavirus* Harbored by Two Sympatric Rodents within the Interior Atlantic Forest

**DOI:** 10.3390/v13101963

**Published:** 2021-09-29

**Authors:** Briana Spruill-Harrell, Anna Pérez-Umphrey, Leonardo Valdivieso-Torres, Xueyuan Cao, Robert D. Owen, Colleen B. Jonsson

**Affiliations:** 1 Department of Microbiology, Immunology and Biochemistry, University of Tennessee Health Science Center, Memphis, TN 38163, USA; bspruill@uthsc.edu; 2 School of Renewable Natural Resources, Louisiana State University and AgCenter, 227 RNR Building, Baton Rouge, LA 70803, USA; aperezumphrey@gmail.com; 3 Department of Microbiology, University of Tennessee-Knoxville, Knoxville, TN 37996, USA; valdivieso.leonardo@gmail.com; 4 Department of Nursing-Acute/Tert Care, University of Tennessee Health Science Center, Memphis, TN 38163, USA; xcao12@uthsc.edu; 5 Centro para el Desarrollo de la Investigación Científica, Asunción C.P. 1371, Paraguay; rowen@pla.net.py; 6 Department of Biological Sciences, Texas Tech University, Lubbock, TX 79409, USA

**Keywords:** hantaviruses, Interior Atlantic Forest, grid enclosure, habitat degradation, species diversity

## Abstract

Understanding how perturbations to trophic interactions influence virus–host dynamics is essential in the face of ongoing biodiversity loss and the continued emergence of RNA viruses and their associated zoonoses. Herein, we investigated the role of predator exclusion on rodent communities and the seroprevalence of hantaviruses within the Reserva Natural del Bosque Mbaracayú (RNBM), which is a protected area of the Interior Atlantic Forest (IAF). In the IAF, two sympatric rodent reservoirs, *Akodon montensis* and *Oligoryzomys nigripes*, harbor Jaborá and Juquitiba hantavirus (JABV, JUQV), respectively. In this study, we employed two complementary methods for predator exclusion: comprehensive fencing and trapping/removal. The goal of exclusion was to preclude the influence of predation on small mammals on the sampling grids and thereby potentially reduce rodent mortality. Following baseline sampling on three grid pairs with different habitats, we closed the grids and began predator removal. By sampling three habitat types, we controlled for habitat-specific effects, which is important for hantavirus–reservoir dynamics in neotropical ecosystems. Our six-month predator exclusion experiment revealed that the exclusion of terrestrial mammalian predators had little influence on the rodent community or the population dynamics of *A. montensis* and *O. nigripes*. Instead, fluctuations in species diversity and species abundances were influenced by sampling session and forest degradation. These results suggest that seasonality and landscape composition play dominant roles in the prevalence of hantaviruses in rodent reservoirs in the IAF ecosystem.

## 1. Introduction

Hantaviruses carried by rodents belong to the genus *Orthohantavirus*, family *Hantaviridae*, within the order *Bunyavirales* [1]. Currently, there are 36 species of *Orthohantavirus* that have been recognized by the ICTV [1], and we denote those recognized by the ICTV as species with the *orthohantavirus* designation. Specific species and genotypes of hantaviruses carried by rodents show a close association with a single reservoir host, which is proposed to be the result of millions of years of coevolution [2,3,4,5]. To date, some hantaviruses carried by rodent reservoirs are associated with human diseases, hantavirus pulmonary syndrome (HPS), or hemorrhagic fever with renal syndrome [6]. Transmission to humans usually occurs through inhalation of aerosolized virus particles from the urine, feces, or saliva of persistently infected rodents [6]. Since the 1993 outbreak of HPS in the southwest USA, cases of HPS have been recognized throughout the Americas with a case fatality rate ranging from 10 to 37% overall [7,8]. Chile, Argentina, and Brazil have the highest number of HPS cases in South America [6]. *Andes orthohantavirus* (ANDV, reservoir *Oligoryzomys longicaudatus*) is the major cause of HPS in Chile and Argentina [9,10] and is the only hantavirus known to be transmitted from person-to-person, albeit limited [11,12,13]. In Brazil, most HPS cases are associated with Juquitiba hantavirus (JUQV, reservoir *Oligoryzomys nigripes*) and Araraquara hantavirus (ARAV, reservoir *Necromys lasiurus*) [14,15,16,17,18].

Currently, there are two hantaviruses associated with cases of HPS in Paraguay and five other viruses for which cases have not yet been identified [19,20,21,22]. *Laguna Negra orthohantavirus* (LNV, reservoir *Calomys laucha*) was responsible for the first outbreak of HPS in western Paraguay in 1995 [20], and a Bermejo-lineage hantavirus (BMJ) was associated with one HPS case in eastern Paraguay in 2005 [19]. Although the reservoir of BMJ was not confirmed for this HPS case, earlier studies identified *Oligoryzomys chacoensis* as a reservoir of this virus [21,22]. In one study, phylogenetic analysis revealed that a BMJ-infected rodent, originally classified as *O. chacoensis* actually belonged to a clade of the *O. flavescens* complex, suggesting that *O. flavescens* is also associated with BMJ or could be the sole reservoir of BMJ (e.g., in the case of taxonomic misidentification), although this warrants further investigation [23]. More recently, Alto Paraguay hantavirus (reservoir *Holochilus chacarius*), first identified in the Chaco in Paraguay [24], was identified in an HPS case in Argentina [25]. The five additional genotypes circulating in Paraguay that have not been associated with cases of HPS therein include Alto Paraguay hantavirus (reservoir *Holochilus chacarius*) in the Chaco, Ape Aime hantavirus (a reassortment, reservoir *Akodon montensis*), Itapúa hantavirus (reservoir *O. nigripes*), Jaborá hantavirus (JABV, reservoir *A. montensis*), and Juquitiba hantavirus (JUQV, reservoir *O. nigripes*); the last four have all been detected in eastern Paraguay [20,22,24,26,27,28]. However, as stated above, JUQV has been associated with numerous cases of HPS in Brazil [14,17,18]. Prior studies suggest substantial levels of hantavirus infection among the Indigeous Aché community of Caazapá Department (Ava’i district) in eastern Paraguay [24]; and in several indigenous tribes in the Chaco of western Paraguay [29,30]; however, the hantavirus(es) responsible for these exposure events remain to be identified.

The prevalence of hantaviruses within rodent reservoir populations is driven by changes in climate and seasonality, food resources (e.g., masting), and/or anthropogenically-driven impacts such as peri-domestic buildings and agricultural landscape structures [31,32,33,34]. These factors can alter the composition of small mammal communities and their predators and interactions, which may have cascading effects on the abundance, behavior, distribution, and movement of hantavirus-infected hosts [35,36,37,38]. Alterations in host-population dynamics can impact the number of intra- and interspecific interactions, thus influencing the number of opportunities for hantaviruses to spread from one host species to another [36,39]. In neotropical ecosystems, the prevalence of hantaviruses in rodents is strongly associated with landscape composition (e.g., amount of land and habitat type) and landscape configuration (e.g., spatial arrangement of elements in the landscape) [28,40,41,42,43,44,45,46,47,48]. Many reservoirs of hantaviruses are habitat generalists and are abundant in human-dominated landscapes such as agricultural fields, pastures, peridomestic habitats, and near forest edges [43,45,49,50,51]. In a country-wide survey of rodents in Paraguay, rodents harboring hantaviruses were more likely to be associated with agricultural landscapes [40]. In the Interior Atlantic Forest (IAF) of eastern Paraguay, an increased prevalence of hantaviruses in rodent reservoirs was detected in forest areas with a moderate level of habitat degradation [47].

Extensive surveillance of rodents in the Reserva Natural del Bosque Mbaracayú (RNBM), a protected area within the IAF, suggests that two hantavirus genotypes are circulating, JABV and JUQV, in two sympatric species, *A. montensis* and *O. nigripes*, respectively. The IAF comprises the western-most portion of the Atlantic Forest, which extends from southeastern Brazil into northeastern Argentina and eastern Paraguay [52]. It is the largest biogeographical sub-region of the Atlantic Forest, covering 72,784,790 ha [53], and it is recognized as one of the world’s 25 biodiversity hot spots, having high biological diversity and endemism [54]. Deforestation caused by agricultural production of soybean, corn, cotton, and cattle has led to the transformation of continuous forest into forest fragments, with less than 7.1% of the original forest remaining today [52,53,55,56].

In prior studies, we have reported that the prevalence of antibody to hantaviruses ranged from 4.4% to 10% in *A. montensis* and 0.02% to 5.6% in *O. nigripes* [26,28,47]. On a macrohabitat scale (i.e., land cover and grid level), *A. montensis* and *O. nigripes* favor areas with human agricultural disturbances and *A. montensis* prefer high-forest habitats [28,40,41]. At the microhabitat level (i.e., trap-station level), *A. montensis* prefer areas with less dense overstory and increased litter (i.e., fallen trees, woody shrubs, and herbaceous plants), while *O. nigripes* have no specific microhabitat preference [28,41,46]. Each species tends to avoid other species within the same area and preferably associate with their conspecifics [57]. We have shown that seropositive *A. montensis* favor areas with denser forest overstory and less litter, have larger home ranges, and have greater longevity than seronegative individuals, suggesting that infection alters the life history strategy of *A. montensis* [46,47].

Recently, we reported field experiments conducted in the RNBM in which experimental sampling grids were provided with supplemental food resources [47]. The addition of resources increased small mammal species diversity; however, it did not affect hantavirus seroprevalence over time [47]. Similar to other studies in the neotropics [40,43,44,51], seroprevalence was primarily driven by landscape and habitat composition; however, this response was not consistent across rodent species. Seropositive *A. montensis* were more likely to be encountered on grids with moderate habitat degradation while habitat did not influence the encountered probability of seropositive *O. nigripes*. This species-specific response reveals the complexity of hantavirus–host dynamics in the neotropics, even among sympatric species, and emphasizes the need to examine other factors that may drive hantavirus prevalence among reservoir populations.

Predation has a major impact on the structure of natural communities and population growth rate of rodents [58]. The reduction of natural predation is one of the most significant effects of anthropogenic action in rural landscapes [59,60]. We hypothesized that predator exclusion would alter species diversity, rodent abundance, and thus the prevalence of hantaviruses among resident rodent reservoir populations. To test this hypothesis, we designed an experiment within the RNBM where three distinct areas of habitat degradation (grids) were enclosed with fencing and predators were removed from the grids, and three similar grid habitats remained unenclosed. The goal of exclusion fencing was to preclude the influence of predation on small mammals on the sampling grids and potentially reduce rodent mortality [61]. A previous study in the RNBM had confirmed that at least eight mammalian carnivore species preyed on sigmodontine rodents there [62], as well as a variety of other native predators which occur there, including didelphid marsupials (opossums; *Didelphis aurita*, *Caluromys lanatus*) and reptiles (*Boa constrictor amarali*; *Oxyrhopus guibei*; *Crotalus durissus, Salvator* spp.) [62,63,64,65]. In addition to the fencing, two types of traps were utilized within the fenced grids to capture and remove all potential predators of small rodents. Herein, we report our findings from this study, which was conducted over an eight-month period. Analysis of the data indicated that the enclosures had no effect on hantavirus seroprevalence in either of the reservoir species, *A. montensis* or *O. nigripes*. Additionally, the results suggest that habitat is the most significant predictor of the seroprevalence of hantavirus in *A. montensis* and *O. nigripes* regardless of the presence or absence of predators.

## 2. Materials and Methods

### 2.1. Study Area

**Figure 1:**
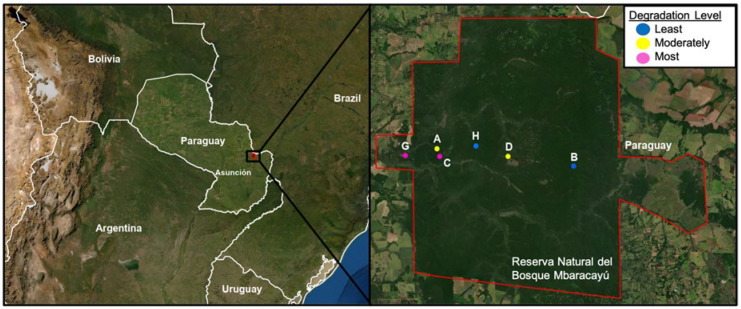
The left panel shows the location of the Reserva Natural del Bosque Mbaracayú (RNBM) in eastern Paraguay near the border with Brazil and is represented with a black box. In the right panel, an enlarged photograph of the RNBM is outlined in red, and the location of the grids is shown. Rodents were sampled from six grids (three enclosed—H, D, C) and three unenclosed—B, A, G). Grids were designated as “least degraded”—B and H, “moderately degraded”—A and D, and “most degraded”—G and C. The image was made using ArcGIS^®^ software by Esri. ArcGIS^®^ and ArcMap™ are the intellectual property of Esri and are used herein under license. Copyright © Esri. All rights reserved.

Sigmodontine rodents were sampled from six grids (three enclosed and three unenclosed) within the Reserva Natural del Bosque Mbaracayú (RNBM) in northeastern Paraguay (Figure 1). The regional climate has marked seasons based on precipitation patterns and is classified as climate type Cfa (temperate, without dry season, hot summer) [66]. October to February is considered the hot, rainy summer season, with February marking the end of the summer with intermediate rainfall levels. May to September is considered the dry, winter season (https://www.meteoblue.com/en/weather, accessed on 20 August 2021). Annual rainfall averages around 1800 mm and annual temperatures range from 14 to 32 °C (www.lacgeo.com/mbaracayu-forest-natural-bioshere-reserve, accessed on 20 August 2021). The RNBM covers an area of approximately 65,000 ha and protects the largest remnant of the Interior Atlantic Forest in Paraguay [67]. The forest is a subtropical, semideciduous forest with an isolated patch of Cerrado (a dry biome of forests, woodlands, and open savannas) in the eastern portion of the reserve. Previously, we measured vegetation structural characteristics at each of the six sampling grids to classify forest degradation and rodent habitat [47]. Grids were designated as “least degraded”—B and H, “moderately degraded”—A and D, and “most degraded”—G and C. Detailed grid descriptions can be found within the Supplementary Text in Camp et al. [47].

### 2.2. Grid Enclosure and Predator Removal

**Figure 2:**
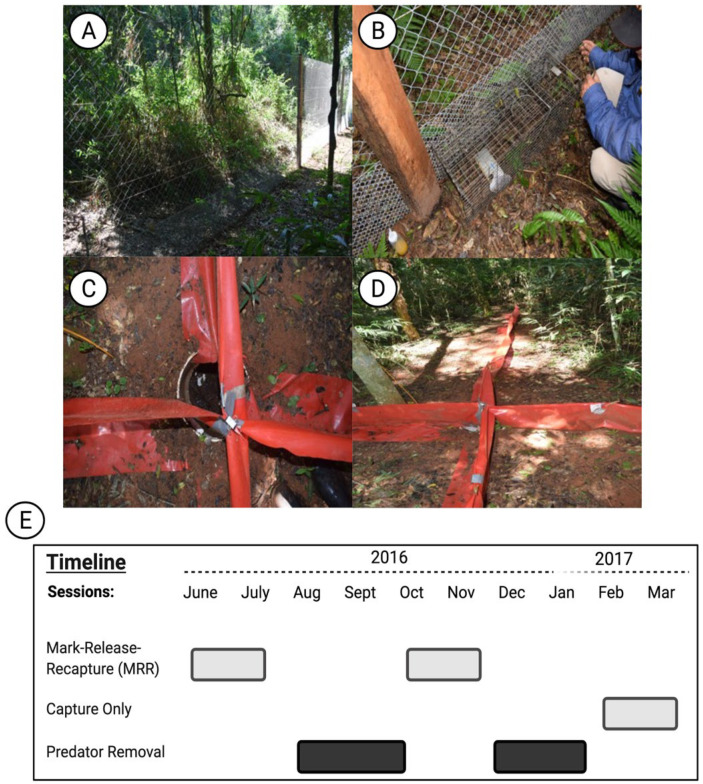
Experimental field design. (**A**) Photo taken of the two-meter-high chain link fencing that was constructed on the three experimental grids—H, D, C. (**B**). Cage trap placed against the inside of the fences, also showing the half-meter high small-mesh chain link fencing. (**C**) Pitfall trap used to capture small terrestrial mammalian predators. (**D**) Image of four 5 m drift fences extending in the four cardinal directions from the pitfall trap mouth. (**E**) Timeline of the study. Pre-treatment sampling (PreTrt) was performed during the winter during June–July 2016. The first post-predator removal session was performed at the beginning of the summer during October–November (ON2016). The final capture only session was performed at the end of the summer during February–March 2017 (FM2017). The timeline image was created with BioRender.com, accessed on 18 September 2021.

Predator exclusion fences were constructed around the three experimental grids—H, D, and C. The fences consisted of two-meter-high chain link fencing of “regular” (5 cm) mesh, plus half-meter high chain link of smaller mesh (2 cm), both buried ca. 10 cm into the ground (Figure 2A). The mesh of the smaller fencing was sufficiently large so that rodents could pass through it, and we observed some rodents passing through it after release. Thus, the fences were presumed not to affect the foraging patterns or other behavioral characteristics of the rodents. The fencing was supported by wooden posts every five meters, and a single gate of ca. 80 cm enabled entry onto the grids by field personnel. In addition, three lines of electric fencing were placed on the outside of the chain link to prevent entry or the re-entry of mammalian predators onto the grid by climbing the fence. Two electric lines were positioned ca. 15 cm from the chain link, at 80 and 120 cm above ground. The third was ca. 5 cm from the chain link, just below the line at 80 cm above the ground. The electric fences were operated with 12-volt batteries, which were replaced each week with a newly charged battery.

These grids were closed immediately after the baseline sampling session in June–July 2016, and remained closed and the electric fences activated, until completion of the final sampling period in February–March 2017. Coincidental to the closure of the grids, we began the predator removal efforts. In each of these, 16 cage traps (Tomahawk or Havahart type) were placed evenly around the border, against the inside of the fence (Figure 2B). These were baited variously with canned sardines, mackerel, or raw meat. In addition, each of these three grids had 16 pitfall traps placed in an “offset uniform” pattern within the grid (uniformly spaced, except offset by 5 m so they did not fall on a rodent trap station). The pitfalls were 50 L hard plastic barrels with screw-on tops; the tops were removed during predator removal sessions and replaced at all other times (Figure 2C). Each pitfall trap had four 5 m drift fences extending in the four cardinal directions from the pitfall mouth (Figure 2D). The predator exclusion sessions were conducted for nine nights each of the months of August, September, October, December 2016, and January 2017, but not in November 2016 or February 2017 when sampling of rodents was being conducted (Figure 2E). During the nine-night sessions, the cage traps were placed for three nights each in each of the three fenced grids, and the pitfall traps were opened for nine nights during each session (although the number of nights varied occasionally). Any potential mammalian or reptilian predators of small mammals that were captured were identified, recorded, marked (generally with a fluorescent spray paint on the back), and either removed from the grid and released or collected. At least one herpetologist accompanied us during each of the predator removal sessions, to search for snakes or predacious lizards, and to identify the reptiles and amphibians captured in the pitfall traps. Finally, to verify the absence of predators within the grid enclosures, camera traps were placed periodically at locations along the inside of the fence, where we expected any mammalian predator would be likely to travel.

### 2.3. Rodent Collection

Sherman traps (7.6 × 8.9 × 22.9 cm, Sherman Trap Company™, Tallahassee, FL, USA) were set at stations 10 m apart in a 12 × 12 grid on each site. Each station had one trap 1–2.5 m above ground in branches or vines and two traps on the ground. Each of the six grids was sampled for five nights during each of the three sampling sessions for a total of 38,880 trap-nights (144 stations × 3 traps/station × 5 nights × 6 grids × 3 sessions). For each capture, the date, grid, station number (row and column), and trap height (on ground or above ground) were recorded. Following the initial pre-treatment baseline survey (June–July 2016 = PreTrt) using a mark–release–recapture (MRR) method, and after approximately three months of predator exclusion, rodents were again sampled with MRR over five nights during October–November 2016 (ON2016), and then sampled again in February–March 2017 (FM2017), after two additional months of predator exclusion (Figure 2E). All captured individuals were collected for tissue harvest in this final sampling session.

Rodents were identified following D’Elía and Pardiñas (2015) and authors therein [68]. The animal’s weight, sex, reproductive status, and age category (juvenile, subadult, adult) were also recorded. Pregnant females and those with an open vagina or developed mammary glands were considered reproductively active, while females with a closed vagina were considered inactive. Males with testicles in the scrotum were considered reproductively active, while males with undescended testicles were considered inactive. Age for each individual species was determined based on weight and pelage. In MRR sampling sessions of July and November of 2016, samples of saliva, blood, urine, feces, and a small (1–2 mm) tail snip were collected, and rodents were individually tagged with a Passive Integrated Transponder (PIT tag) and released at the point of capture. In the February 2017 sampling, rodents were taken to the field laboratory, where liver, lung, heart, kidney, muscle, spleen, colon, blood, urine, saliva, and embryos (when encountered) were harvested and stored immediately in liquid nitrogen, prior to shipping to the University of Tennessee Health Science Center, where the samples were stored at −80 °C until processing. Clinical signs, such as enlarged spleen, liver parasites, or tail lesions or scars from *Leishmania* spp. infections, were recorded during necropsy. At present, all voucher specimens are held in an authorized collection (Paraguayan Secretaria del Ambiente, Habilitación No. 004/2015, approved 15 October 2015) of Robert Owen.

All voucher specimens will be deposited in the Museo Nacional de Historia Natural del Paraguay, or another accredited Paraguayan research collection. Tissue samples not consumed from this research and other ongoing projects will be deposited with the Natural Science Research Laboratory at the Museum of Texas Tech University.

### 2.4. Hantavirus Antibody Screening by Immunofluorescence Assays (IFA)

Blood samples (*n* = 632 unique specimens) from all rodent species were collected across the three different sampling sessions and screened for the presence of antibodies cross-reactive with antigens from ANDV by indirect immunofluorescence assay (IFA) [24]. Antigen was prepared by acetone fixation of Vero E6 cells infected with ANDV on 10-well spot slides. Blood was diluted 1:32 in phosphate buffer saline (PBS) and incubated with antigen in duplicates at 37 °C for 30 min. After incubation, secondary antibody, Alexa Fluor 488 F (ab’)2 Fragment rabbit anti-mouse IgG (H + L) (Invitrogen Cat. No. A21204), was added to each well to identify antibody-positive rodents by fluorescent microscopy.

### 2.5. Statistical Analyses

All statistical analyses were performed in R version 3.6.3 [69]. The study was a 2 × 3 complete block design with repeated measures: two treatments (control, enclosure) grouped into three degradation levels (each representing a block), which were measured at three time points. We took the experimental design into consideration for all subsequent analyses except where noted. The raw data used for analyses are available in the Supplementary Material file: Predator Database. 

Rodent abundance was estimated for each individual species (*A. montensis*, *O. nigripes*, and *H. megacephalus*), at each grid for each MRR session using Huggins closed-capture models [70,71] in Program MARK v 6.2 [72]. Closed-capture models assume that there are no animal turnovers due to births, deaths, and movement on or off grids. Given the short number of sampling nights per session (5 nights) compared to the lifespan of rodents [73], we considered the population closed. Capture and recapture probabilities were modeled as constant (M_0_), time varying (M_t_), and behavior varying (M_b_). The best candidate model for each session was chosen based on Akaike’s information criterion (AIC_c_) with adjustment for small sample size. With the derived estimates, we fitted linear mixed effects models to test whether abundance (dependent variable) differed by treatment, degradation level, and session (fixed effects; R package lme4) [74]. Grid was treated as a random effect. We did not include interactions to avoid overfitting given the small sample size.

Rodent species diversity was calculated using the Gini–Simpson Index (1-*D*) (R, package Vegan version 2.5–6) [75]. Values range from 0 (least diversity) to 1 (maximal diversity) and are derived using the equation: 1−Σp_i_^2^, where p_i_ = the total number of individuals in species i divided by the total number of species [76]. This metric assigns more weight to the most common species and is interpreted as the probability that any two species selected at random from all individuals in a community will be different species [76]. We selected 1-*D* as a measure of species diversity because we were less interested in rare species and *Akodon montensis*, *Hylaeamys megacephalus*, and *Oligoryzomys nigripes* are the most abundant at our study sites [28,47]. As with the abundance estimates, we fitted the data to linear mixed effects models to test the effects of treatment, session, and degradation level on rodent species diversity, treating grid as a random effect.

Seroprevalence was calculated as the number of individuals with antibodies that cross-reacted with ANDV as determined by IFA, divided by the total number of blood samples tested. Univariate logistic regression analyses were used to assess the impact of age, sex, weight, reproductive status, and the presence of a tail scar (evidence of *Leishmania* infection) on seropositivity (R package, logistf) [77]. We fitted the logistic model using Firth’s bias reduction method to correct for small sample sizes [78]. Univariate logistic regression was also used to examine the association of treatment, session, and degradation level on the presence of antibodies to hantaviruses for each individual species. Due to the limited number of seropositive captures for each species, we did not consider the recapture and blocking structure of grids.

### 2.6. Ethics Statements

All animal procedures were approved (Approval No. 14024-03, ACUP No. 18-108) by the Texas Tech University Institutional Animal Care and Use Committee (IACUC), which follows the 8th Edition of the Guide for the Care and Use of Laboratory Animals (Guide), NRC 2011, and the Animal Care and Use Committee guidelines of the American Society of Mammalogists for the use of wild mammals in research and education. The study did not involve endangered or protected species. 

## 3. Results

### 3.1. Rodent Sampling

A total of 1026 rodent captures were recorded during sampling, including 695 individuals and 331 recaptures. Thirty of the recaptures were encountered in a session different from their initial capture resulting in 725 unique session captures (Table 1). These 725 individuals were used in subsequent analyses unless otherwise noted. For example, for IFA screening, only 632 unique blood specimens were available for testing. Overall, these captures belonged to 12 native rodent species belonging to the family Cricetidae, subfamily Sigmodontinae. Species richness (i.e., the total number of species) varied from two to seven on each grid (Table 1). The most common species captured were *Akodon montensis* (63.3%), *Hylaeamys megacephalus* (16.6%), and *Oligoryzomys nigripes* (10.3%).

### 3.2. Population Size, Abundance, and Experimental Variables

**Figure 3:**
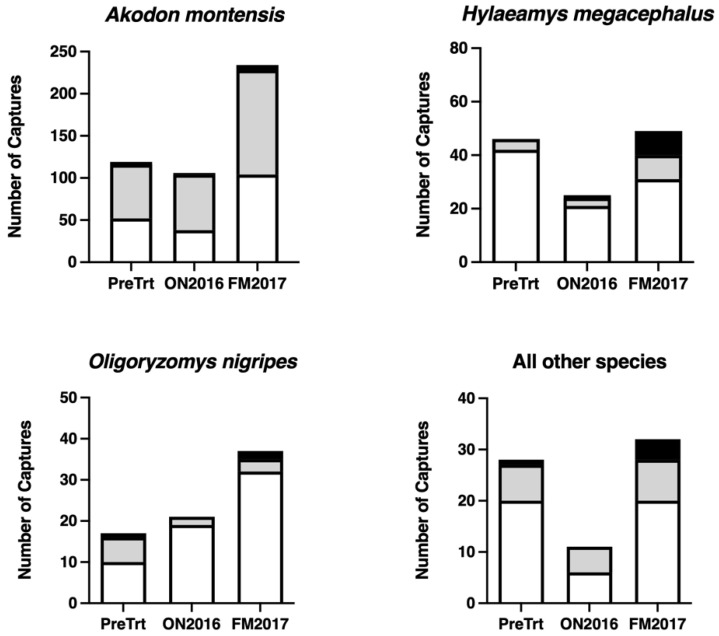
The number of unique individuals per session by age class for *Akodon montensis*, *Hylaeamys megacephalus*, *Oligoryzomys nigripes*, and all other species. Juveniles are shaded in black, subadults are shaded in light gray, and adults are shown in white. Pre-treatment sampling (PreTrt) was performed during the winter during June–July 2016. The first post-predator removal session was performed at the beginning of the summer during October–November (ON2016). The final capture only session was performed at the end of the summer during February–March 2017 (FM2017).

We evaluated population size for the three most common species, *A. montensis*, *H. megacephalus*, and *O. nigripes*. All species displayed similar seasonal patterns in population sizes with higher capture numbers at the end of the summer session (FM2017, Figure 3). *H. megacephalus* and all species other than *A. montensis*, *H. megacephalus*, and *O. nigripes* showed a decline in population size from the winter (PreTrt) to the early summer session (ON2016), while *A. montensis* and *O. nigripes* exhibited more stable population sizes (Figure 3). Adults and subadults were captured more frequently than juveniles (Figure 3). However, for *H. megacephalus* and *O. nigripes*, there were comparable numbers of juveniles and subadults in the FM2017 session (Figure 3). We found no statistical difference in the capture ratio of male and female unique individuals (data not shown).

Rodent abundance estimates were derived using Huggins closed-capture models where capture and recapture probabilities did not vary (M_0_ models) and where capture probabilities varied by trapping day (M_t_ models) and behavior (M_b_ model; Table S1). For *A. montensis*, recapture probabilities were higher than initial capture probabilities in the ON2016 session, indicating a “trap happy” response (Table S1). We did not detect a significant effect of sampling session on the average abundance of *A. montensis* or *O. nigripes* (Tables S2 and S3). However, the abundance of *H. megacephalus* was significantly lower in the ON2016 session (M = 5.46) compared to the PreTrt session (M = 11.3; *p* = 0.0377; Table S3), confirming the differences observed for *H. megacephalus* population size (Figure 3). We did not detect an effect of grid enclosure or forest degradation level on the average abundance of *A. montensis*, *H. megacephalus*, or *O. nigripes* (Tables S2–S4).

### 3.3. Effects of Grid Enclosure, Session, and Forest Degradation Level on Rodent Species Diversity

To test whether species diversity (1-*D*) differed by grid enclosure, session, or degradation level, we used a linear mixed effects model as described in the *Statistical Analyses* section. Results indicated that forest degradation level but not treatment or session had a marginal effect on species diversity. Grids with moderate (*β* = 0.178, SE = 0.049, *p* = 0.0689) and the most degraded (*β* = 0.212, SE = 0.049, *p* = 0.0504) forests had higher diversity compared to the least degraded forests (Table 2).

### 3.4. Hantavirus Seroprevalence

Of the 632 blood specimens available for testing, blood was analyzed from 602 individuals and 30 recaptures. These recaptures were encountered in a session different from their initial capture. We detected antibodies that were cross-reactive with ANDV antigen from the blood of two species, *A. montensis* and *O. nigripes*. Both species are known reservoirs of hantaviruses (JABV and JUQV, respectively) [26,28,47]. *O. nigripes* had the highest seroprevalence, with 10 of 66 (15.2%) individuals seropositive, while *A. montensis* were 3.5% seropositive (Table 3). We identified two *A. montensis* and two *O. nigripes* that seroconverted during the study. For the two species combined, seroprevalence was highest in the early summer session (ON2016, 6.5%) and on grids with the most degraded forests (6.6%, Table 3).

Using univariate logistic regression, we examined the association of age, sex, weight, reproductive condition, and the presence of a tail scar (evidence of *Leishmania* infection) with the presence of hantavirus antibodies (Table 4). Due to a limited number of seropositive captures, we did not consider the recapture and blocking structure of grids as effect factors. Weight (*n* = 3) and tail scar data (*n* = 1) were missing from four *A. montensis* in addition to weight data from three *O. nigripes*. Therefore, these individuals were excluded from further analyses. For *A. montensis*, we found that age, sex, weight, reproductive condition, and the presence of a tail scar were all associated with being seropositive, whereas sex and weight were associated with *O. nigripes* seropositivity (Table 4).

Seropositive *A. montensis* were more likely to be heavier, male adults with a tail scar. Specifically, males were 4.5 times more likely to be seropositive (*p* = 0.0144) than females, adults were 3.8 times more likely (*p* = 0.0176) to be seropositive than subadults, reproductively active individuals (as described in the methods) had higher odds (3.6 times, *p* = 0.0240) of being infected, and individuals with a tail scar were 6.1 times more likely (*p* = 0.0012) to be infected (Table 4). For each 1 g increase in weight, there was a 13.5% increase in the odds of an individual having antibodies to hantaviruses (*p* < 0.0001, Table 4).

For *O. nigripes*, seropositive individuals were heavier males (Table 4). No juveniles were identified as seropositive. Sex was a strong predictor of seroprevalence because only males were seropositive: the odds of being seropositive were 14.7 times higher for males than females (*p* = 0.0083). For each 1 g increase in weight, there was a 26% increase in the odds of an individual having antibodies to hantaviruses (*p* = 0.0109, Table 4).

### 3.5. Effects of Grid Enclosure, Session, and Forest Degradation Level on Rodent Seroprevalence

To test the effect of grid enclosure, session, and degradation level on the prevalence of antibodies to hantavirus for each reservoir species (*A. montensis* and *O. nigripes*), we used univariate Firth’s logistic regression disregarding the block design structure and recapture of 30 rodents (Table 5). We observed a significant association of seropositivity with forest degradation level in *A. montensis* but not in *O. nigripes*. *A. montensis* individuals captured in the most degraded forests were 7.58 times more likely to be seropositive then those captured in the least degraded forests (95% CI = 1.71–71.41, Table 5). We did not observe the association of seropositivity with treatment or sampling sessions in either *A. montensis* or *O. nigripes* (*p* > 0.1, Table 5).

### 3.6. Effects of Species Diversity on Rodent Seroprevalence

Given that we observed a marginal association of rodent species diversity with forest degradation level for *A. montensis* but not *O. nigripes*, we used univariate Firth’s logistic regression, disregarding the block design structure and recapture of 30 *A. montensis* and *O. nigripes* to test two hypotheses that have been generally proposed in the literature regarding the relationship between species diversity and pathogen prevalence: the “dilution effect” and the “amplification effect” [79]. For directly transmitted pathogens such as hantaviruses, the “dilution effect” posits that in a highly diverse community, (1) non-reservoir species may “dilute” pathogen prevalence by reducing the probability of encounters with other reservoirs or (2) interspecies competition for food resources could reduce the abundance of reservoirs [79,80]. In contrast, prevalence may be “amplified” in communities with high species diversity if interspecies competition is minimal and reservoir abundance increases [79]. We did not observe an association of seropositivity with species diversity in *A. montensis* (*p* = 0.1728) or in *O. nigripes* (*p* = 0.9766, data not shown).

## 4. Discussion

In the past century, numerous previously unknown RNA viral pathogens have emerged and reemerged with an estimated frequency of one new pathogen every 18 months [81,82,83]. Of these new pathogens, the majority have zoonotic origins in wildlife, are widely distributed in nature, and most are RNA viruses. Some of the RNA viruses harbored by rodents that are associated with geographically localized outbreaks of human diseases include a number of New Word hantaviruses (e.g., SNV, ANDV, ARAQV) and arenaviruses (*Chapare mammarenavirus*, *Lassa mammarenavirus* (LASV), and *Argentinian mammarenavirus* (formerly Junin virus (JUNV))) [11,84,85,86,87,88,89]. The increased recognition of the global prevalence and burden of new and reemerging strains of hantaviruses and arenaviruses alone presents challenges in global public health. Although many principles have been derived from past epidemics and pandemics, there remain major gaps in our understanding of the ecology and natural history of zoonotic viruses carried by rodents, e.g., what are the potential reservoirs for direct or sylvatic transmission, how they are maintained in their reservoirs, and what processes and mechanisms lead to spillover, transmission, and sustained transmission in human populations [81]. For most of the viruses circulating in South America, we have only seen the “tip of the iceberg” in terms of these critical questions. For example, in South America, there are more than 400 species of sigmodontine rodents [68], of which fewer than 100 have been tested for hantaviruses [16,90], and few have been tested for the presence of other viruses.

Longitudinal field studies have provided a wealth of knowledge regarding the natural ecology of the rodent reservoirs of hantaviruses across the globe, e.g., [6,46,91,92,93,94,95]. Extrinsic drivers that correlate with an increase in the prevalence of hantaviruses harbored by reservoir rodents include climate and/or landscape change [40,41,96]. In North America, particularly in the southwest, outbreaks of HPS cases correlate with weather and climatic events, especially precipitation [92,96,97,98,99]. In our research in South America [22,26,27,40,41,46,100,101,102,103,104,105,106,107,108,109,110] as well as that of others in Central America (e.g., [14,42,44,111]), a strong correlation exists between landscape [14,42,44,111,112] and viral prevalence in rodent populations. Analyses of the community ecology of the host rodent community in Paraguay demonstrate that the composition of the rodent community changes spatially in response to land cover variation, as well as temporally (both seasonally and interannually) [46,113,114]. To further address how hantaviruses harbored by rodents are maintained in the neotropics, and what additional local factors may drive changes in virus prevalence, we have designed experimental field studies to probe two key ecological factors: predation (herein) and increased food availability [47]. In the recent report of our experimental field studies to evaluate the effects of resource augmentation [47], we noted a change in rodent species community composition, although with no discernable effect on the prevalence of hantavirus (antibodies) in the reservoir hosts (*A. montensis* and *O. nigripes*) over time, nor was there evidence of a dilution effect [47]. We also noted that the habitat composition at two spatial levels, independent of resource addition, is a primary driver of the prevalence of hantaviruses in the neotropics [47]. In these new experimental field studies reported herein, we sought to address how reduction of predation would affect rodent community structure and the presence of hantavirus in the reservoir rodents. This study and the prior study were conducted in a reserve in the IAF in isolated areas with no human movement except our field team and occasional Indigenous hunters, who did not enter the enclosed grids (Figure 1).

As briefly discussed in the introduction, the RNBM is a protected area with high diversity in plant and mammal populations. The predator community in the reserve is exceptionally diverse, represented by six mammalian families [62,115], as well as several reptile species (snakes, lizards, and caiman). In field studies, understanding how multiple predators impact the rodent community and individual species is logistically challenging. However, several groups have attempted to address this by (1) examining the foraging patterns and behaviors of rodents following exposure to predator odors (e.g., urine and feces) [116,117,118,119] and (2) experimentally altering predator abundance through live trapping or exclusion by barrier fencing [120,121,122]. Our predator exclusion fencing and trapping protocols were thorough in removing and excluding reptile and mammalian predators of small mammals. Their effectiveness was verified by periodic photographic sampling (camera traps) of likely mammal trails and by monthly trapping of mammals and extensive searches by professional herpetologists. We were unable to prevent activity by avian predators, but experience suggests that this is negligible in the heavily forested areas where our grids were placed, due to heavy vegetative cover.

We were unable to measure an effect of either predator exclusion or forest degradation on the abundances of the two hantavirus reservoirs, *A. montensis* and *O. nigripes*. Other studies have reported inconsistent results of the effects of predator exclusion on rodent densities. In some systems, predator abundance was not related to rodent population abundance [123], and in others, predator abundance was only related to the abundance of specific rodent species. For example, a 7-year study in native grasslands of western Montana, USA, found that predator exclusion did not affect the abundance of deer mice (*Peromyscus maniculatus*), which is the reservoir of SNV. However, following the exclusion of both generalist and specialist predators, the abundance of montane voles (*Microtus montanus*) increased [122]. Where predator presence was found to affect rodent prey abundance, reports have indicated that the effects may be complexly interrelated with habitat, existing rodent densities, season, individual species behavioral characteristics, and type of predator [122,124,125,126]. These potential interactions are difficult to tease apart, both conceptually and statistically, and their resolution will require intensive long-term studies in natural and managed field conditions.

Given the block design without replications (six grids), we were unable to statistically examine the interaction between degradation level and predator exclusion on rodent abundance. As previously mentioned, evidence suggests that habitat may be interrelated with predator presence [117,118,120,127]. In the Atlantic forests of Paraguay and Brazil, *A. montensis*, a primarily terrestrial species, is associated with dense ground vegetation and open canopy cover [28,41,46,128,129,130,131] while in Brazil, *O. nigripes*, an arboreal species, is associated with denser understory vegetation (e.g., shrubs) and lower canopy cover [128,129,130], suggesting that these microhabitat associations may be preferred as a function of predator avoidance. When examining the independent effect of forest degradation level on rodent abundance, there were no differences in the average abundances of *A. montensis*, *O. nigripes*, or *H. megacephalus* (Tables S2–S4). These species were captured in all habitat types, although the abundance of *O. nigripes* was higher on moderately and most degraded grids compared to least degraded grids, and the abundance of *A. montensis* was lower on moderately and most degraded grids compared to least degraded grids. It is noteworthy to mention that each grid pair was defined broadly into three categories based on the dominant vegetation type. The least degraded grids were associated with more deadwood (fallen branches) on the ground, higher canopy, and a shorter distance to the nearest tree, and the most degraded grids were associated with the presence of forbs, logs (fallen trees), and orange trees (*Citrus aurantium*, a non-native species that has acclimatized to the forest) [47]. Although we did not examine the microhabitat (i.e., station-level) associations for each species in this study, we previously reported that *A. montensis* was positively associated with fallen branches, while *O. nigripes* was not associated with any particular microhabitat characteristic [47]. *A. montensis* use fallen branches for shelter and nesting, which could provide an explanation to why more rodents were found on the least degraded grids [132].

Overall, our results were consistent with other studies given that *O. nigripes* and *A. montensis* are not vulnerable to habitat fragmentation [128,133]. Historically, rodent community structure within the neotropics has been evaluated across different habitat types (e.g., native forests, pastures, agricultural fields) of different sizes, edge densities (length of the forest edge in contact to non-forest), connectivity (area of the fragment plus the area connected to the fragment by corridors of natural vegetation), and percent forest cover (e.g., [40,43,49,133,134,135,136,137]). *A. montensis* and *O. nigripes* have been identified in agricultural fields (e.g., eucalyptus plantations), rural buildings, and native forest areas [133,134,137]. These species are especially abundant at forest edges, agricultural fields, and on small, isolated fragments where species richness and diversity is typically low [134,137]. However, species richness and abundance have been shown to be greater on smaller fragments that have an increased edge effect relative to larger ones [49]. Although our study sites (grids) were within native forest areas and the forest degradation level of each grid was quantified using the mean vegetation characteristics at each trap station (i.e., macrohabitat characteristics), the ability of these species to maintain stable populations on each grid irrespective of forest degradation level may be an important factor influencing the capacity of these rodents to occupy agriculturally disturbed landscapes [40,134].

It is important to note that the seasonal dynamics of rodent populations may have restricted our ability to examine predator exclusion-induced effects on rodent abundance. Seasonality is considered an intrinsic characteristic shaping the dynamics of rodent populations [124]. We observed an increase in population sizes of *A. montensis*, *O. nigripes*, and *H. megacephalus* in the FM2017 session (Figure 3), although, we did not compare rodent abundance by treatment in the FM2017 session given that this was a capture-only session. Huggins’ closed-capture models consider both capture and recapture probabilities to provide reliable estimates of rodent abundance; therefore, the study design limited abundance calculations by this method [70]. While the logistics of sampling replicate grids and habitat types longitudinally is daunting, future studies would benefit from increased number of grids and increased monthly sampling to examine the relationships between seasonality, rodent abundance, and predation.

We found a marginal association between forest degradation level and species diversity, which is a finding different from what we have found in earlier studies in the RNBM [47]. The relationship was non-monotonic, in that the most degraded grids had higher species diversity than either the least or moderately degraded grids and the moderately degraded grids had higher species diversity than the least degraded grids. We were unable to demonstrate an effect of predator exclusion on species diversity nor an association of species diversity with seroprevalence. Interestingly, *A. montensis* seropositivity was significantly higher on the most degraded grids compared to the least degraded grids, suggesting that most of the degraded forests appear to amplify hantavirus seroprevalence. Similar to our study, Milholland et al. [138] observed that rodent communities with higher species diversity (regardless of habitat) can have higher hantavirus seroprevalence compared to communities with low species diversity. It was suggested that communities with higher species richness can maintain hantavirus infection through spillover within assemblages of closely related species [138]. Although we did not observe any spillover occurrences, we previously reported spillover of JUQV in *O. mattogrossae* (originally reported as *O. fornesi*) [22,24,27], and others have detected spillover of JABV in two closely related species, *Akodon paranaensis* and *A. serrensis* in Brazil [139,140,141].

Results of our analyses herein confirmed earlier conclusions [47] that seroprevalence is strongly associated with individual characteristics (sex, age, weight, reproductive status, Leishmania infection) in both hantavirus reservoirs in our study area (*Akodon montensis* and *Oligoryzomys nigripes*).

There are strong theoretical and practical reasons to hypothesize that predator abundance would indirectly affect viral prevalence in rodent reservoirs [37,142,143]. By reducing reservoir host abundance, predators may decrease viral prevalence within rodent communities. Nevertheless, we did not find that predator exclusion affected seroprevalence in either of the reservoir species. However, for *A. montensis*, we found that forest degradation level, not species diversity or predator exclusion, was independently associated with the presence of hantavirus antibodies. Individuals captured on the most degraded forest grids were more likely to be seropositive than those captured in the least degraded grids. These findings were consistent with our previous studies where we found that the odds of seropositivity were higher on moderately and the most degraded grids compared to the least degraded grids [47]. In this study, seropositive *A. montensis* were more likely to be found on moderately degraded grids compared to the least degraded grids; however, this did not reach statistical significance (Table 5). Previously, we reported that having antibodies to hantavirus altered the microhabitat association of *A. montensis* [47]. At the microhabitat level, seropositive *A. montensis* are less likely to be found in their predicted habitat niche (i.e., areas with increased litter) and are more likely to be found in habitats with a high percentage of grasses and no fallen trees or orange trees (i.e., native, undisturbed forest), suggesting that infection could alter the life-history strategies of these species [47]. Collectively, our field experiments confirm that landscape composition (e.g., amount of land and habitat type) plays a predominant role in *A. montensis* population dynamics and hantavirus prevalence. In conclusion, the overall environment of neotropical natural systems of the rodent community (including both habitat quality and predation) exerts complex effects on both the rodent species and their zoonotic viruses.

## Figures and Tables

**Table 1:** Summary of the unique captures per grid and session.

	PreTrt						ON2016						FM2017						Total
Grid	B	A	G	H	D	C	B	A	G	H	D	C	B	A	G	H	D	C	
Enclosure	−	−	−	−	−	−	−	−	−	+	+	+	−	−	−	+	+	+	
Predator Removal	−	−	−	−	−	−	−	−	−	+	+	+	−	−	−	+	+	+	
Degradation Level	Least	Moderate	Most	Least	Moderate	Most	Least	Moderate	Most	Least	Moderate	Most	Least	Moderate	Most	Least	Moderate	Most	
Species																			
*Akodon montensis*	27	28	16	21	7	20	13	18	12	18	15	30	42	61	18	48	10	55	**459**
*Calomys callosus*	0	0	0	0	0	1	0	0	0	0	0	0	0	0	0	2	0	0	**3**
*Euryoryzomys russatus*	0	0	0	0	0	0	0	0	0	1	0	1	0	0	0	0	0	0	**2**
*Hylaeamys megacephalus*	4	12	7	4	6	13	1	7	3	5	5	4	7	15	2	7	8	10	**120**
*Juliomys pictipes*	1	0	0	0	0	0	0	0	0	0	0	0	1	0	0	0	0	0	**2**
*Nectomys squamipes*	0	0	0	0	0	0	0	0	0	0	0	0	0	0	1	0	0	0	**1**
*Oecomys mamorae*	0	0	0	0	0	0	0	1	0	0	0	0	0	0	1	0	0	0	**2**
*Oligoryzomys flavescens*	0	2	2	0	0	0	0	0	0	0	0	0	0	3	0	0	0	2	**9**
*Oligoryzomys mattogrossae*	4	2	3	0	1	9	0	0	0	0	0	0	1	7	4	3	0	3	**37**
*Oligoryzomys nigripes*	3	6	3	0	0	5	2	1	5	1	5	7	4	14	3	0	3	13	**75**
*Scapteromys aquaticus*	0	0	0	0	0	0	0	0	0	0	0	0	0	0	0	0	1	0	**1**
*Sooretamys angouya*	1	0	1	0	0	1	2	0	3	0	1	2	0	1	1	0	0	1	**14**
**Total**	**40**	**50**	**32**	**25**	**14**	**49**	**18**	**27**	**23**	**25**	**26**	**44**	**55**	**101**	**30**	**60**	**22**	**84**	**725**
Species Richness	6	5	6	2	3	6	4	4	4	4	4	5	5	6	7	4	4	6	

**Table 2:** Linear mixed effects model with treatment, session, and degradation level as fixed effects and species diversity (1-*D*) as the dependent variable. Results show model coefficient estimates (*β*), the standard error of those estimates (SE), and associated *p* values.

Effect ^a^	Level	Estimate (*β*)	SE	*p*
Treatment	Yes	−0.043	0.040	0.3950
Session	ON2016	−0.040	0.044	0.3884
	FM2017	−0.045	0.044	0.3343
Degradation Level	Moderately	0.178	0.049	0.0689
	Most	0.212	0.049	0.0504

^a^ For each effect, the reference group for each level are the levels not shown. The “Treatment” effect level is in reference to the control (unenclosed) grids. The “Session” effect level is in reference to the “PreTrt” session. The “Degradation level” effect is in reference to the least degraded grids.

**Table 3:** Hantavirus seroprevalence by sampling session and degradation level.

		*Akodon montensis*		*Oligoryzomys nigripes*		Total	
Variable	Level	Pos/Tested	% Pos	Pos/Tested	% Pos	Pos/Tested	% Pos
Session ^a^	PreTrt	2/100	2.0%	2/13	15.4%	4/167	2.4%
	ON2016	5/74	6.8%	3/20	15.0%	8/124	6.5%
	FM2017	7/228	3.1%	5/33	15.2%	12/341	3.5%
Degradation Level	Least	1/146	0.7%	1/8	12.5%	2/190	1.1%
	Moderately	4/126	3.2%	3/27	11.1%	7/216	3.2%
	Most	9/130	6.9%	6/31	19.4%	15/226	6.6%
	Total	14/402	3.5%	10/66	15.2%	24/632	3.8%

^a^ Pre-treatment sampling (PreTrt) was performed during the winter during June–July 2016. The first post-predator removal session was performed at the beginning of the summer during October–November (ON2016). The final capture only session was performed at the end of the summer during February–March 2017 (FM2017).

**Table 4:** Univariate logistic regression tests for the association of age, sex, weight, reproduction condition, and tail scar with the presence of antibodies to *Andes orthohantavirus*. Outputs of each model are the odds ratio, 95% confidence intervals (CI), and associated *p* values. We also report the number of seropositive and seronegative individuals for each predictor level and the proportion of individuals at each level (expressed as a percentage).

Species	Predictor	Level	No. Neg (%)	No. Pos (%)	Odds Ratio ^a^	95% CI	*p*
*A. montensis*	Age	Adult	175 (94%)	11 (6%)	3.847	1.251–15.297	0.0176
		Juvenile	8 (100%)	0	3.454	0.025–40.153	0.4877
		Subadult	205 (99%)	3 (1%)			
	Sex	Male	205 (94%)	12 (6%)	4.465	1.314–23.063	0.0144
		Female	183 (99%)	2 (1%)			
	Weight ^b^		388 (6~63)	14 (14~59)	1.135	1.083–1.196	<0.0001
	Reprod. Condition	Active	68 (92%)	6 (8%)	3.578	1.195–10.251	0.0240
		Inactive	320 (98%)	8 (2%)			
	Tail Scar	Yes	135 (92%)	11 (8%)	6.123	1.988–24.367	0.0012
		No	252 (99%)	3 (1%)			
*O. nigripes*	Age	Adult	46 (82%)	10 (18%)	4.290	0.473–569.134	0.2358
		Juvenile	1 (100%)	0	6.333	0.029–1443.403	0.4150
		Subadult	9 (100%)	0			
	Sex	Male	33 (77%)	10 (23%)	14.731	1.742–1928.789	0.0083
		Female	23 (100%)	0			
	Weight ^b^		56 (6~30)	10 (19~31)	1.264	1.052–1.577	0.0109
	Reprod. Condition	Active	28 (87.5%)	4 (12.5%)	0.692	0.175–2.540	0.5796
		Inactive	28 (82%)	6 (18%)			
	Tail Scar	Yes	1 (50%)	1 (50%)	5.842	0.440–78.007	0.1620
		No	55 (86%)	9 (14%)			

^a^ For each predictor level, odds ratios are in reference to the last level listed. ^b^ weight ranges in grams are listed following the number of seronegative/seropositive animals.

**Table 5:** Univariate logistic regression tests for the association of grid enclosure, session, and degradation level with the presence of antibodies to *Andes orthohantavirus*. Outputs of each model are the odds ratio, 95% confidence intervals (CI), and associated *p* values.

Species	Predictor ^a^	Level	Odds Ratio ^a^	95% CI	*p*
*A. montensis*	Treatment	Yes	0.679	0.198–1.981	0.4901
	Session	ON2016	3.118	0.728–17.749	0.1270
		FM2017	1.334	0.349–7.239	0.6907
	Degradation Level	Moderately	3.563	0.649–35.88	0.1488
		Most	7.584	1.713–71.41	0.0054
*O. nigripes*	Treatment	Yes	0.692	0.175–2.540	0.5796
	Session	ON2016	0.920	0.153–6.285	0.9272
		FM2017	0.888	0.182–5.514	0.8877
	Degradation Level	Moderately	0.714	0.097–8.211	0.7552
		Most	1.275	0.213–13.624	0.8046

^a^ For each predictor, the reference group for each level are the levels not shown. The odds ratio for “Treatment” is in reference to the control (unenclosed) grids. The odds ratio for “Session” is in reference to the “PreTrt” session. The odds ratio for “Degradation level” effect is in reference to the least degraded grids.

## Data Availability

The data presented in this study are available in the supplementary material file: Predator Database.

## Supplementary Materials

https://www.mdpi.com/article/10.3390/v13101963/s1, Table S1: The best-supported Huggins closed-capture models by species, Table S2: *Akodon montensis* abundance, Table S3: *Hylaeamys megacephalus* abundance, Table S4: *Oligoryzomys nigripes* abundance.
