# Supplementary material for: Impact of Predator Exclusion and Habitat on Seroprevalence of New World Orthohantavirus Harbored by Two Sympatric Rodents within the Interior Atlantic Forest
Source: Viruses. 2021 Sep 29;13(10):1963. doi: 10.3390/v13101963 (PMC8538774; doi:10.3390/v13101963)
Supplement: Supplementary file 1 [file viruses-13-01963-s001.zip › viruses-1368889-supplementary.pdf]

Supplementary Tables

Table S1. The best-supported Huggins closed-capture models by species.

Species	Session	Model[a]	AICc	AICc Weight	Parameters	Deviance	(-)2Log(L)
Akodon montensis	PreTrt	M_t	698.8093	0.99627	5	760.7257	688.7074
	ON2016	M_b	711.8354	0.49619	2	653.0708	707.8127
Hylaeamys megacephalus	PreTrt	M_0	259.4307	0.68924	1	237.7558	257.4131
	ON2016	M_0	150.4944	0.67143	1	131.3512	148.4618
Oligoryzomys nigripes	PreTrt	M_t	110.3562	0.44100	5	84.2635	99.5967
	ON2016	M_0	109.9696	0.72171	1	100.9016	107.9241
All other species	PreTrt	M_t	1240.6837	1.00000	5	1453.8901	1230.6264
	ON2016	M_0	1056.2051	0.64236	1	1051.001	1054.2

[a]Capture and recapture probabilities were modeled as constant (M0), time varying (Mt), or behavior varying (Mb). These models were ranked by the AICc. Pre-treatment sampling (PreTrt) was performed during the winter from June-July 2016. The first post – predator removal session was performed at the beginning of the summer in October – November (ON2016).

Table S2. *Akodon montensis* abundance. Linear mixed effects model with treatment, session, and degradation level as fixed effects and abundance as the dependent variable. Results show model coefficient estimates (β), the standard error of those estimates (SE), and associated p values.

Effect[a]	Level	Estimate (β)	SE	*p*
Treatment	Yes	-0.708	7.752	0.9355
Session	ON2016	-3.184	4.564	0.5165
	FM2017	-3.272	9.495	0.7633
Degradation Level	Moderately	-0.392	9.495	0.9709
	Most	-0.708	7.752	0.9355

[a]For each effect, the reference group for each level are the levels not shown. The "Treatment" effect level is in reference to the control (unenclosed) grids. The "Session" effect level is in reference to the "PreTrt" session. The "Degradation level" effect is in reference to the least degraded grids.

Table S3. *Hylaeamys megacephalus* abundance. Linear mixed effects model with treatment, session, and degradation level as fixed effects and abundance as the dependent variable. Results show model coefficient estimates (β), the standard error of those estimates (SE), and associated p values.

Effect[a]	Level	Estimate (β)	SE	*p*
Treatment	Yes	0.656	3.265	0.8592
Session	ON2016	-5.803	2.068	0.0377

Effect[a]	Level	Estimate (β)	SE	*p*
	FM2017	5.642	3.999	0.2937
Degradation Level	Moderately	4.738	3.999	0.3578
	Most	0.656	3.265	0.8592

[a]For each effect, the reference group for each level are the levels not shown. The "Treatment" effect level is in reference to the control (unenclosed) grids. The "Session" effect level is in reference to the "PreTrt" session. The "Degradation level" effect is in reference to the least degraded grids.

Table S4. *Oligoryzomys nigripes* abundance. Linear mixed effects model with treatment and degradation level as fixed effects and abundance as the dependent variable. Session was not modeled as a fixed effect because in the PreTrt session, there were no captures on two out of the three experimental grids. Results show model coefficient estimates (β), the standard error of those estimates (SE), and associated p values.

Effect[a]	Level	Estimate (β)	SE	*p*
Treatment	Yes	0.860	2.148	0.7277
Degradation Level	Moderately	1.934	2.631	0.5387
	Most	3.868	2.631	0.2793

[a]For each effect, the reference group for each level are the levels not shown. The "Treatment" effect level is in reference to the control (unenclosed) grids. The "Session" effect level is in reference to the "PreTrt" session. The "Degradation level" effect is in reference to the least degraded grids.
